# Role of the Primate Perirhinal Cortex in Memory and Emotional Regulation: Ontogeny and Early Insults

**DOI:** 10.18103/mra.v14i2.7272

**Published:** 2026-02

**Authors:** Jocelyne Bachevalier, Alison R. Weiss

**Affiliations:** 1 Emory National Primate Research Center, Atlanta, GA, USA; 2 Oregon National Primate Research Center, Beaverton, OR, USA

**Keywords:** Medial temporal lobe, recognition memory, working memory, executive functions, epilepsy, Alzheimer’s disease, schizophrenia

## Abstract

The perirhinal cortex, a small strip of the anterior medial temporal cortex, first came into prominence through studies of memory. While examining patients with damage to the medial temporal lobe as well as animals with similar regional damage, findings showed that combined damage to the hippocampus, amygdala, and adjacent cortical areas, including the perirhinal cortex, were responsible for the profound memory loss observed. Later, however, the evidence demonstrated that the accompanying damage to the underlying medial temporal cortical areas were largely responsible for the memory deficit that had been attributed to the combined hippocampal and amygdala lesions. The perirhinal cortex has become appreciated as a critical structure supporting familiarity judgement, recognition memory, flexible executive control and behavioral regulation. The objective of this article is first to review the anatomy of the perirhinal cortex and its interactions with other medial temporal structures as well as the neocortex. A series of neurosurgical ablation studies in nonhuman primates will provide evidence for its role in memory and behavioral regulation in adulthood. The next section will highlight the functional maturation of the perirhinal from infancy through adulthood and will show that its role in support of recognition memory emerges in early infancy. The findings will also show that the neonatal perirhinal dysfunction results in functional compensation of recognition and working memory in adulthood but impacts higher-order executive processes such as cognitive control and flexibility. Interestingly, the discovery of the role of perirhinal cortex in familiarity judgements instead of recollection, which is mediated by the hippocampus, is now well documented in several clinical neurodevelopmental disorders (epilepsy, Alzheimer’s disease, schizophrenia), providing valuable markers in the prodromal phase of the diseases for early diagnosis and treatment.

## Introduction

**Figure 1:**
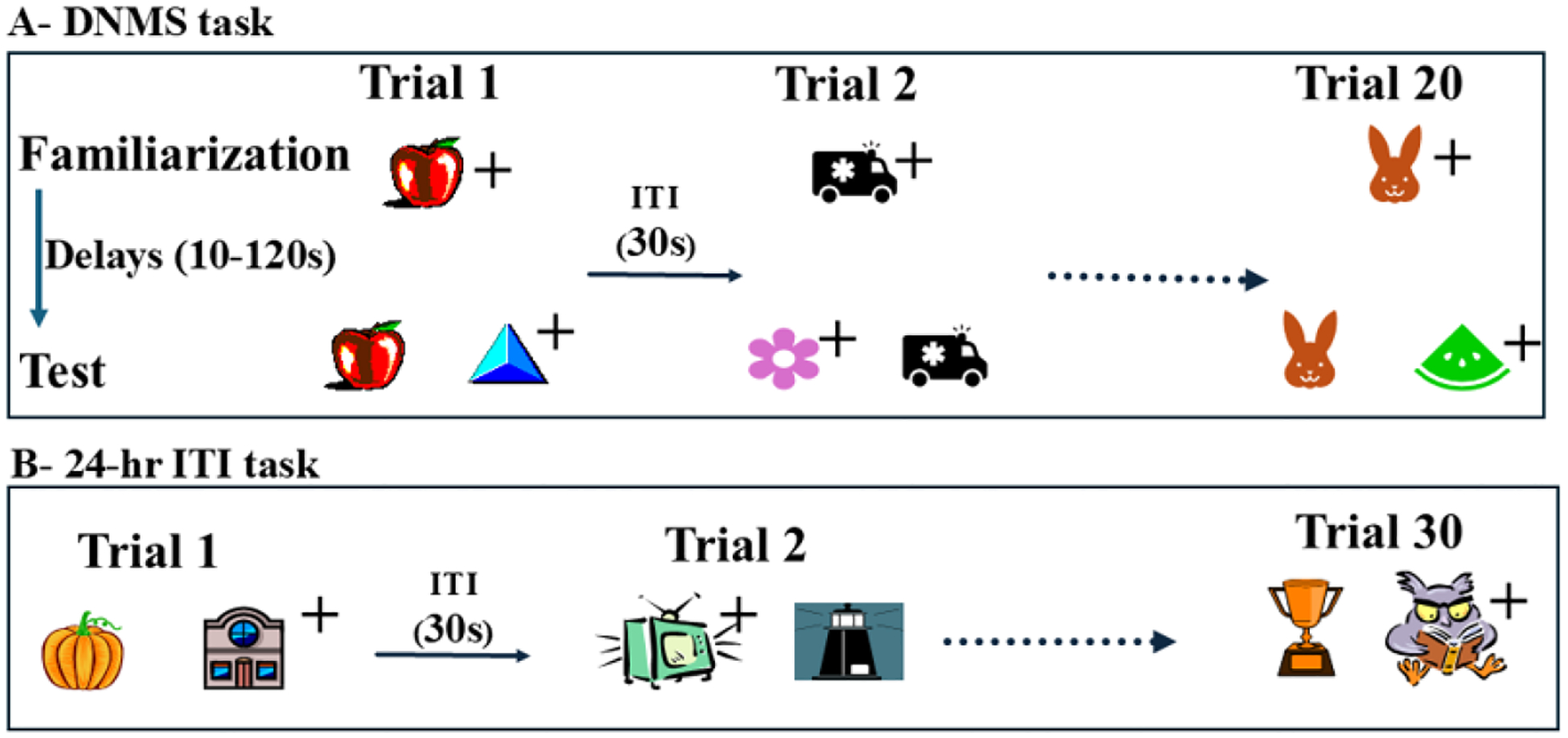
Schematic representation of the cognitive tasks: A- Delayed Non-Match to Sample (DNMS) task measures object recognition. For each trial, animal is first presented with a rewarded objects (apple) and after a delay (10s to 120s), the familiar and a new object (blue triangle) are presented side-by-side and animal must select the new object. Animals received 20 trials/day, each trial using a new set of objects on each trial until reaching the criterion of 90 percent corrects/day. Then, they received a performance test of 100 trials each in which the delays were increased from 30, 60 and 120 sec followed by 100 trials in which the list of familiar objects to remember increased from 3, 5, 10 objects. B- 24-hr ITI (intertrial interval) task measures procedural learning. On each day, animals are presented with a list of 30 discrimination problems in which one of the 2 objects is rewarded. The list is presented once a day until the animals reached a criterion of 90% correct. On both tasks, + indicates the rewarded objects.

**Figure 2:**
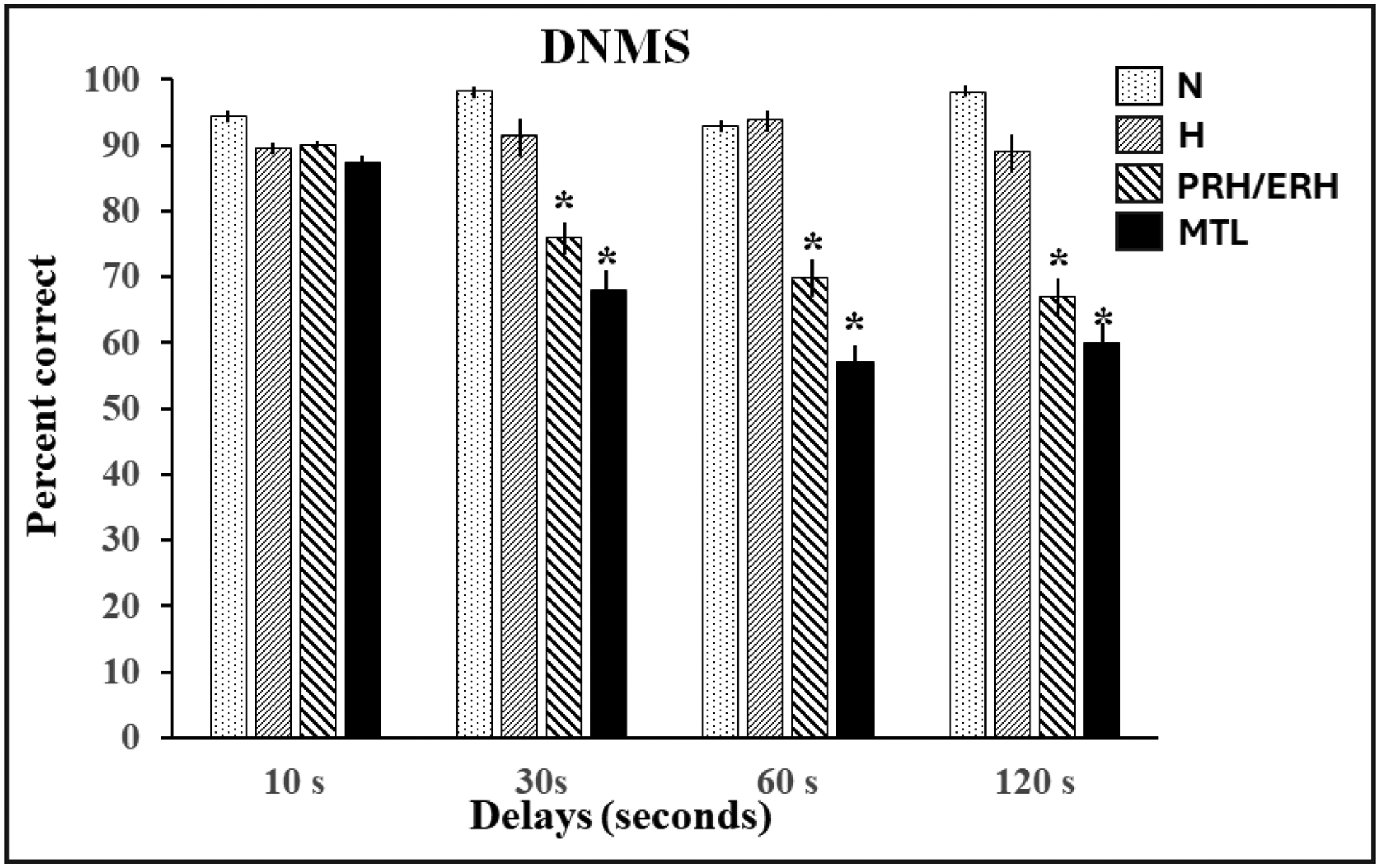
Delayed nonmatching-to-sample (DNMS): Percent correct at four delays (10, 30, 60 and 120 seconds) for normal adult controls (N, n=4), monkeys with hippocampal lesions (H, n=3), monkeys with combined perirhinal/entorhinal lesions (PRH/ERH, n=7) and monkeys with lesions including the amygdala, and adjacent cortical areas (MTL, n=3). SEM is indicated by vertical lines and * Indicates p<.05 compared to N. Data for Groups N, H, and MTL are from Mishkin (1978) and for group PRH/ERH from Meunier et al (1993).

Declarative memory enables access to information previously encoded and stored in long-term memory and involves processes such as recognition and recall. This memory system is distinguished – functionally and anatomically – from procedural, implicit, habit memory systems, which expresses previously acquired skills through performance. Substantial progress was made in the last half century to further our understanding of the neural substrates involved in each of these memory systems. The discovery of the crucial role of the medial temporal lobe in human declarative memory, and the numerous subsequent monkey studies that this discovery prompted, has been the main driving force of research on recognition memory in primates. Hence, we chose to keep the present review focused on the medial temporal lobe. Human patients with large extensive damage to this region suffer global anterograde amnesia in that they are unable to recognize or recall events even after a few minutes^1–3^. Yet, this profound memory deficit left patients’ skill learning and memory abilities intact, leading to the view that the medial temporal lobe supports declarative memory. The modular organization of memory processes was replicated in nonhuman primates following bilateral medial temporal damage originally reported in the amnesic patients, which included two deep structures – the amygdala (AMY) and hippocampus (HIP)- wrapped rostrally by two medial temporal cortical areas, the entorhinal (ERH) and perirhinal (PRH) shown below. Monkeys with medial temporal lobe lesions failed to recognize objects a minute or so after they were presented when tested with the delayed nonmatching-to-sample task (DNMS, Figures 1A and 2), compared to normal intact control groups^4^.

Despite the profound recognition deficit, they were still able to learn long lists of 30 pairs of objects (in which only one of the objects of the pair was rewarded) as rapidly as normal animals even though this list was presented only once every 24 hours (24-hr ITI task, see Figure 1B). Although damage to the medial temporal lobe region in monkeys replicated some of the memory loss seen in the amnesic patients, the results from DNMS task were soon called into question when more selective lesion techniques made it possible to parse out the individual contributions of specific medial temporal lobe structures to recognition memory. Although aspiration amygdala lesions in adult monkeys had no effects on recognition memory at any delays, restricted damage to the subjacent PRH/ERH cortex devastated animals’ performance even at the shortest delay^5,6^. Hippocampal damage resulted at best in a mild impairment only at longest delays of 10 minutes and beyond^7–11^. Taken together, these data indicated that the impaired recognition memory observed after the large medial temporal lobe lesions likely resulted from damage to the medial temporal cortical areas and not from damage to the amygdala or hippocampus. Given the anatomical organization of the region, it is currently being argued that the PRH may be important for visual perception of complex items as well as memory (see review^12^ for a more detailed discussion). Given the prominent impacts these new findings had on the neural bases of memory processes and on the neural underpinning of clinical neurodevelopmental disorders, this manuscript reviews the discoveries made in the last decade. Following a brief summary of the anatomical organization of the PRH cortex, we describe its functional organization in adult primates demonstrating the modulation of its interactions with the hippocampus to support recognition memory as well as its interactions with the AMY to reduce anxiety in presence of familiar positive stimuli. Next, the postnatal morphological maturation of the PRH cortex as well as the developmental trajectory of PRH recognition memory functions will illustrate that the PRH cortex functionally matures earlier after birth than the hippocampus in monkeys. The long-lasting effects of neonatal PRH damage on memory functions will then be briefly described. The review will end with an account of the implication of PRH dysfunction to clinical neurodevelopmental disorders, including epilepsy, schizophrenia, and Alzheimer’s Disease (AD).

## Primate Perirhinal Cortex Anatomy and Functional Organization

**Figure 3:**
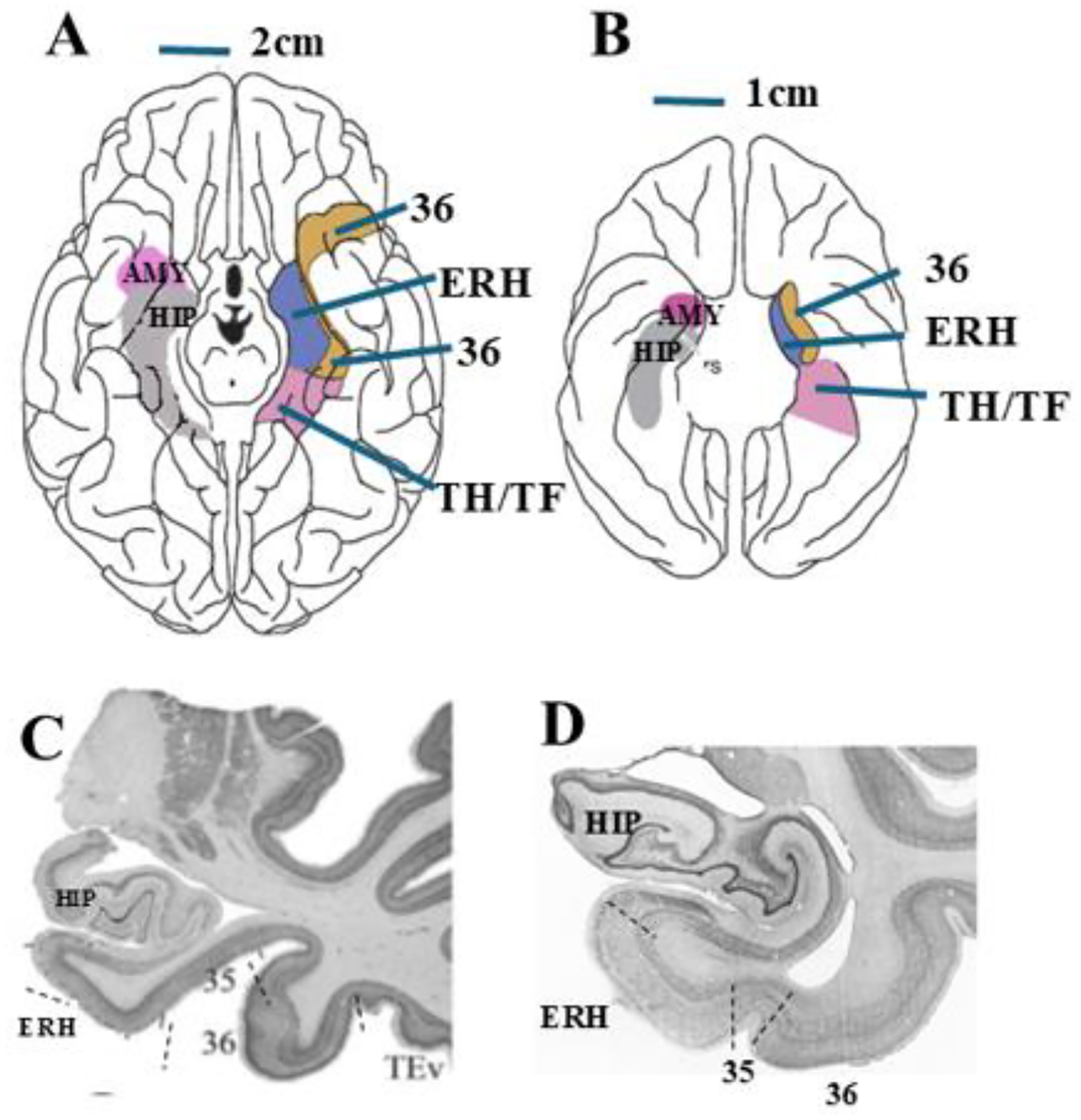
Perirhinal cortical areas of the human and monkey brains. Drawings of the ventral view of the human (A) and monkey (B) brains, depicting the location of the perirhinal cortex (area 36; area 35 buried within the rhinal sulcus and not shown), entorhinal cortex (ERH) and parahippocampal areas TH/TF, on the right side and the amygdala (AMY) and hippocampus (HIP) on the left side. Histological coronal sections through the anterior temporal lobe, depicting the entorhinal cortex (ERH), perirhinal areas 35 and 36 and hippocampus (HIP) in humans (C) and macaques (D). Dashed lines indicate cytoarchitectural borders between cortical areas. Rs: rhinal Sulcus. Illustrations in A and B are adapted from Murray et al.^193^.

In both humans and monkeys, the PRH is a narrow band of cortex lying lateral to the collateral and rhinal sulcus, respectively, in the anterior portion of the medial temporal lobe (Figure 3A–D). The PRH, defined by Brodman areas (BA) 35 and 36^13^, receives prominent and convergent inputs from sensory areas (visual, somatosensory, and auditory), polymodal areas (cingulate cortex, superior temporal sulcus, and parahippocampal gyrus), reward-related cortical areas (orbitofrontal cortex) as well as interconnections with other MTL structures (hippocampus and amygdala)^14–18^, but its densest afferent inputs originate in ventral visual cortical region (temporal areas TE and TEO)^19^. In turn, the PRH projects to the anterior and lateral portions of the ERH, with lighter projections to the distal section of CA1 and the proximal section of the subiculum of the hippocampus^14–16,20^. It also projects to the lateral amygdala nuclei^21^ and has bi-directional monosynaptic projections with the lateral prefrontal fields (BA 45, 47/12, 46, 9)^22^. Multi-synaptic pathways through the thalamus connect the PRH to the medial prefrontal areas (Brodmann’s areas 24, 25, 32, and 14)^19,23,24^. In sum, its convergent inputs from sensory, polymodal, and reward-related cortical areas together with its prominent interconnections with other medial temporal lobe and prefronl cortex structures place it in a unique position to coordinate neural representations of non-spatial visual information generated in the ventral visual stream with brain structures known to be important for memory (hippocampus), executive functions (prefrontal cortex) and emotional regulation (amygdala). Given these widespread connections with other brain structures, the PRH cortex has been implicated in diverse functions that are summarized next.

### Perirhinal Cortex and Visual Perception

Neurocognitive processes underlying visual perception begin in peripheral sensory organs (eyes), where visual stimuli are transduced into neural signals. Basic information about color, movement and form are coded and assembled into progressively complex representations, first in primary visual cortex (areas V1-V4) and then in the higher-order visual cortical areas of the dorsal and ventral visual streams. Seminal work demonstrated that dorsal visual areas in inferior parietal cortex allowed integration of visuospatial features to represent locations, whereas the ventral visual areas in inferior temporal cortex allowed integration of perceptual features to represent objects^25–30^. It is in the PRH, the last station of the object visual pathway, that a complete representation of a perceived object is realized^14,16,31,32^. In this way, the PRH may generate complex representations of objects from a compilation of perceptual features represented at lower levels of the visual hierarchy.

Most of the knowledge on the PRH role in perception comes from a body of work that has used neuroimaging and electrophysiological recording techniques to monitor PRH activity during perceptual tasks or tested visual discrimination abilities after PRH damage. Neuroimaging data in healthy human adults indicate that the PRH is highly active during perceptual tasks requiring the integration of visual features into configurally-based representations. This pattern of activation corresponds to the activity in ventral temporal visual areas that have been historically linked to visual processes^33^. Additionally, when subjects performed difficult figure-ground discrimination problems, the activity recorded in area V2 mimicked the activity usually recorded in PRH^34^. Taken together, these findings suggest that the PRH may interact with other cortical areas important for feature-based visual processing to construct visual representations from configurations of familiar features. Thus, it was not surprising to observe that PRH lesions resulted in visual discrimination impairment when stimulus complexity was high or perceptual overlap between stimuli was extensive (i.e. feature ambiguity), although they spared the ability to discriminate two highly dissimilar stimuli^32,35–40^. The specificity of the visual deficits following PRH lesions gave credibility to the neuroimaging data suggesting the importance of this cortical area in mechanisms of complex visual perception^31^.

### Perirhinal Cortex and Familiarity Judgments

The PRh may not only be critical for object perception, as its strong anatomical link with the ERH and hippocampus has led scientists to believe that it may also be critical for memory processes such as recognition. Recognition of a previously encountered item can be accomplished by two processes: either recollecting the specific episode in which it was previously encountered, or by assessing the degree of its familiarity. In this way, mechanisms of recollection and familiarity are both capable of supporting recognition memory. Lesion studies in rodents and monkeys^5,6,41–44^, as well as work with human neuropsychiatric populations^45–47^ have all demonstrated the importance of the PRH for recognition memory. Yet, newer studies have begun to provide evidence for the presence of a functional dissociation in the contribution of the PRH and hippocampus to recognition memory, with the PRH being critical for familiarity judgments and the hippocampus associated with recollection. For instance, patients with extensive medial temporal lobe damage encompassing both hippocampus and PRH show deficits in recollection and familiarity judgments^47^. However, selective damage to the hippocampus, sparing the PRH, impairs recollection but spares familiarity^48,49^. Furthermore, electrophysiological recordings have demonstrated that neuronal firing in the PRH precedes cell firing in the hippocampus, suggesting that there is a rapid familiarity signal mediated by the PRH, which is then followed by a later-onset recollection signal mediated by the hippocampus^50,51^. Additional neuroimaging studies in healthy adults have shown that hippocampal activity increases in response to retrieval of information but not in response to judgments of familiarity^52,53^. In sum, the PRH appears to be important to support familiarity processes involved in recognition memory (see for additional details^54^).

### Perirhinal Cortex and Working Memory

The PRH is also involved in the integration of multiple sensory features, across multiple domains, into an abstract view-invariant representation of a stimuli and is critical for familiarity recognition of these stimuli; in this fashion, the strong projection of the PRH with the lateral prefrontal cortex could enable memory processes that have been linked to this area, such as working memory. Working memory involves the maintenance of a limited set of cognitive representations of objects, places, ideas, goals, or rules. Furthermore, these cognitive representations are kept active in a manner flexible enough to cooperate with simultaneous/parallel working memory process that monitor or manipulate the representations being kept ‘in mind^55–61^. In the last 50 years, overwhelming evidence has accumulated from human functional imaging^55–57,59^ and electrophysiological and lesion studies in monkeys^58,60–66^ to indicate the importance of the ventrolateral prefrontal cortex for maintenance processes and the dorsolateral prefrontal cortex for higher-order monitoring/manipulation processes. However, more recent studies suggest that the prefrontal cortex is part of a broader network of interconnected brain areas involved in working memory^67^. Specifically, medial temporal lobe structures are also recruited during working memory tasks^68–73^, such as the PRH cortex that is well positioned to play a role in working memory, mainly because of its direct reciprocal connections with lateral and orbital prefrontal cortex fields^14,15,19,24^.

Electrophysiological and functional imaging studies indicate increased activity in PRH during object-based working memory tasks, suggesting that it supports object representations used by the prefrontal cortex during working memory. Specifically, cells in the macaque PRH are highly activated during working tasks requiring the temporary maintenance of object representations, but this activity is not observed in other temporal visual areas such as area TE^74^. Likewise, 2-Deoxyglucose imaging studies demonstrate increased PRH activity during a delayed object alternation task requiring the maintenance and monitoring of information, whereas the same increase was not seen in the ERH^68^. Taken together, these results point to a unique contribution of the PRH to performance on tasks that require the active/flexible representation of familiar items, that is strengthened by the concurrent lack-of-contribution of both its primary afferent inputs (area TE/TEO) as well as its primary efferent projections to ERH.

### Perirhinal Cortex and Proactive Interference

Proactive interference occurs when previously acquired information impedes the ability to learn or apply new information and may result in behavior that is dominated by rules no longer appropriate to the current situation^75–77^. To resolve proactive interference, the influence of formerly active, and now competing, response sets must be suppressed^78,79^. This requires the inhibition of behavioral responses based on “old” information, and a flexible shift of cognitive resources towards learning/remembering “new” information^78^. These cognitive mechanisms are critical for performance on tasks requiring participants to flexibly update cognitive representations or shift response strategies, such as the Wisconsin card sort task, the self-ordered pointing task, and attentional set-shifting task^80–82^. Lesion studies in monkeys have already demonstrated that behavioral inhibition is supported by the orbital frontal cortex, whereas cognitive flexibility is supported by the ventrolateral and medial prefrontal cortex^79,83–86^. Given that the PRH has robust interconnections with these prefrontal areas^14,15,19,87,88^, its contribution to mechanisms underlying cognitive flexibility and/or behavioral inhibition is expected. There is already evidence that combined damage to PRH and ERH in adulthood impairs performance on reversal-learning tasks, suggesting that the PRH plays a role in mechanisms of behavioral inhibition^89,90^. In contrast, preliminary results on the effects of extended medial temporal lobe damage in adulthood indicates that the medial temporal lobe structures are not important to support performance on attentional set shifting tasks^91^. However, few studies to date have directly addressed the role of the PRH in mechanisms of cognitive flexibility in adulthood, or during development (but see below in “Early perirhinal cortex functions and dysfunction”) and additional information on this topic will be valuable.

### Perirhinal Cortex and Emotional Regulation

As reviewed above, recent efforts to define the functions of the primate ERH and PRH areas have focused on their interactions with the hippocampus in the mediation of normal memory. Its implication in affective functions through, for example, its dense connections with the amygdala, has received little attention despite supporting experimental^92^ and theoretical^93^ evidence from the rodent literature. In an electrophysiological study simultaneously recording neurons in PRH, ERH, and the basolateral amygdala nucleus (BLA) during a trace-conditioning task, Paz, Pelletier, Bauer and Pare^94^ reported that early in the learning process, BLA activity was associated with increased signal transmission from PRH to ERH, and this activity was increased markedly after reward delivery. In this way, the PRH appears to serve as an active gateway of information flow from neocortical areas toward the hippocampus, which can be modulated by the emotional salience of the present situation provided by the amygdala^95,96^. To provide additional evidence for this proposal, we first explored the role of the primate PRH, by comparing the behavioral responses of monkeys with combined lesions of the PRH and ERH with those of monkeys with neurotoxic or aspiration amygdala lesions^97^. Four stimuli probed affective functions (construed in a broad sense to encompass social behavior, emotion, and motivation). Two of these stimuli had a social component (an unfamiliar human and a conspecific stimulus) and two were nonsocial items, one affectively positive (a generally rewarded object) and one affectively negative (a toy snake). A detailed ethogram was built to quantify behaviors during three weekly presentations of each stimulus. In this paradigm, combined ERH/PRH ablations yielded none of the symptoms recorded after lesions involving the amygdala; i.e. hyperorality (a tendency to explore any items by mouth), and hypermetamorphosis (compulsive eating, hypersexuality, excessive affiliation, and diminished fear). Rather, they led to more subtle behavioral changes that were opposite in direction, namely, the ERH/PRH lesions reduced affiliative responses and heightened defensiveness, indicating that ERH and PRH damage can interfere with responses to affectively salient stimuli in a way radically opposite from those following amygdala damage. A follow up study investigating the effects of separate lesions of the ERH and PRH in the same paradigm^98^ showed similar emotional changes to those of the combined lesions (i.e., attenuated affiliation and enhanced defense). Although failure to modulate responses based on previous experience (i.e., memory difficulties) may explain these affective changes, it does not account, however, for the sparing of some memory-dependent modulations of defense, nor for the lack of correlation between the animals’ affective changes and their own recognition memory performance. Alternatively, we had proposed that damage to ERH and PRH introduces a negative bias in the risk assessment of affectively salient stimuli; a proposal more compatible with Gray and McNaughton’s^93^ anxiety-centered view of medial temporal functions, than with prominent mnemonic/perceptual functional models of the hippocampus-PRH duo. To sum, the PRH could modulate the interactions between the hippocampus and the amygdala to reduce anxiety in presence of familiar positive stimuli.

## Early Perirhinal Cortex Functions and Dysfunction:

**Figure 4:**
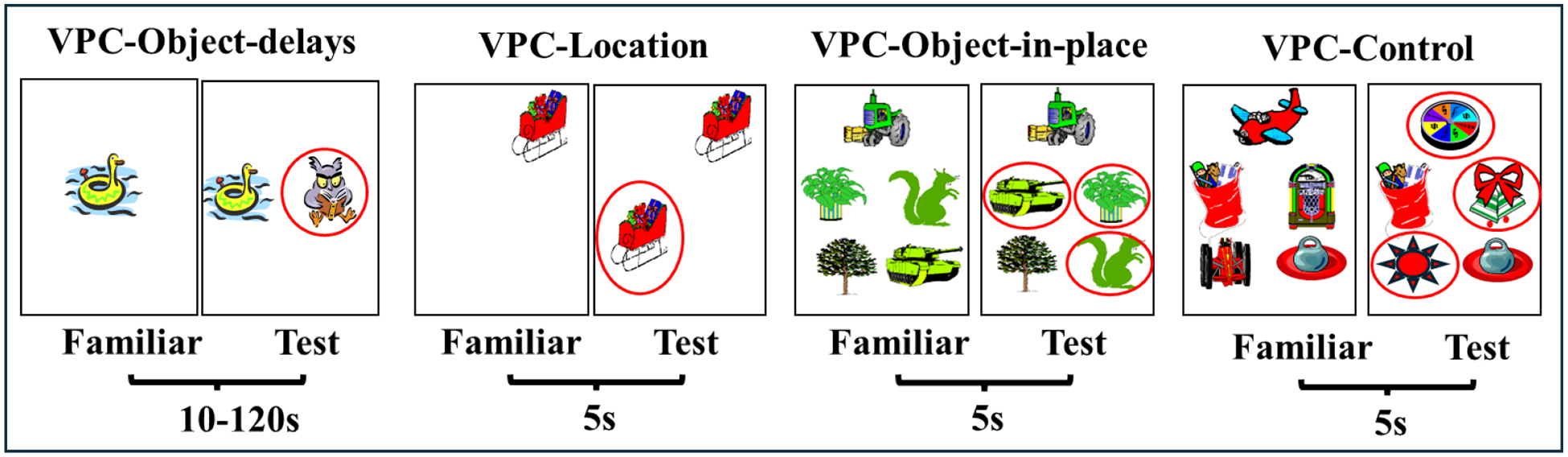
Visual Paired Comparison (VPC) tasks measure incidental object and spatial recognition memory. For VPC-Object-delays, the comparison was between a single familiar object and a novel object. For the VPC-Location, the comparison was between a single object in a spatial location of the screen and a duplicate object in a new location. For the VPC-Object-in-Place, measuring object-place associations, the comparison was between the array of five objects and the same objects with permuted locations of three of the objects for the new picture. For the VPC-Control, the comparison was between an array of five objects and the same array with three of the objects replaced with new objects. Familiarization time was 30s for all tasks. Modified from Bachevalier (2019).

As reviewed above for adult monkeys, in addition to its role in building representations of objects, the role of PRH in object recognition memory has received growing support from studies in several species including rodents, monkeys and humans. Yet, its role in the maturation of early memory functions had not been explored in human infants or infant monkeys. However, this was made possible with the exploitation of another paradigm, the visual paired comparison task (VPC) that enabled evaluation of incidental recognition memory skills in nonverbal infant primates of both species. Thus, we began a series of prospective developmental studies to assess the role of the hippocampus and PRH in the maturation of recognition memory using the VPC-Object-delays task (Figure 4). In this task, monkeys are first familiarized to view an object at the center of a monitor and after a delay their recognition memory was indexed by longer looking time to novel stimuli as compared to familiar ones. Earlier studies had already shown that incidental recognition-memory ability is present as early as 3–4 days of age with either no delays or 2 min delays^99^ and become stronger in 3-month-old human infants^100^. Similarly, by 4 weeks of age, infant macaques show novelty preference that becomes stronger by 13 weeks of age^101^.

### Neonatal Perirhinal Lesions and Object and Spatial Memory Development

To gain additional knowledge on the developmental trajectory of recognition memory abilities from birth to adolescence and its neural substrate, we longitudinally tested sham-operated controls (Neo-C) and those with neonatal lesions to either the PRH cortex (Neo-PRH) or the hippocampus (Neo-HIP, for comparison) from 1 to 48 months, using the 4 VPC tasks^102–105^ shown in Figure 4. All lesions were performed between 10–12 days of age. The pattern of performance in the age-matched controls as well as the memory impairment following the two types of lesions was surprising, but at the same time very revealing.

#### Visual recognition memory:

**Figure 5:**
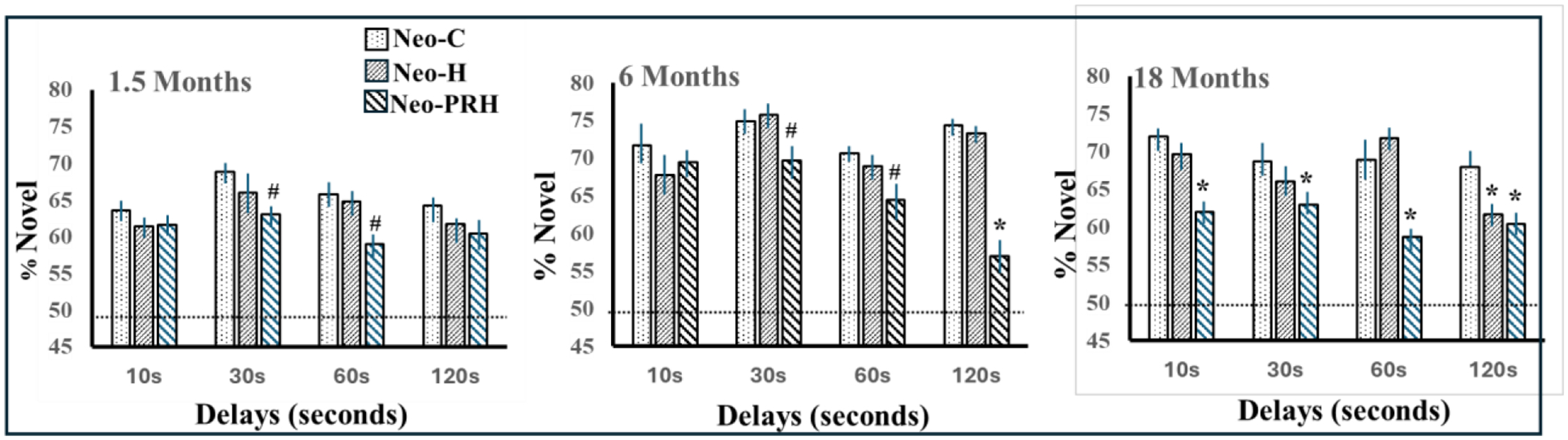
VPC-Object-delays task: Percent looking at novel image after delays of 10s, 30s, 60s, and 120s at 1.5, 6 and 18 months in sham-operated monkeys (Neo-C), monkeys with neonatal hippocampal lesions (Neo-H) and monkeys with neonatal perirhinal lesions (Neo-PRH). Chance is at 50%. * Indicates p<.05 and # indicates p<.06 compared to Neo-C. Data are from Zeamer et al. (2010, 2015).

The development of object recognition abilities was assessed in infant monkeys from 1.5 to 18 months with the VPC-Object-delays task. Normally developing monkeys showed robust recognition memory across short and long delays, with a delay-dependent forgetting emerging only at 18 months of age^102^ (Figure 5) that was similar to that shown in adult monkeys^106^. Interestingly, infants with Neo-HIP lesions performed as well as controls at the 2 youngest ages, but, at 18 months, showed a significant forgetting that became evident and reliable only at the longest delay of 120s (Figure 5), suggesting that, with maturation, animals with neonatal hippocampal lesions grew into a recognition memory loss that remained present even when reaching adulthood^107^.

Both the emergence of delay-dependent recognition memory performance at 18 months of age in the control animals together with the recognition memory loss observed after neonatal hippocampal lesions at that same age suggest that important maturational changes in the neural substrate supporting incidental recognition memory occurred after six months of age in monkeys. This interpretation was supported by anatomical findings indicating that the primate hippocampus is not fully developed at birth and go through significant anatomical remodeling until at least 1–2 years of age in monkeys^108–110^. By contrast, these object recognition memory abilities were severely compromised in infant monkeys with neonatal PRH damage early after birth^103^ (Figure 5). This memory loss emerged after 1.5 months of age but became more pronounced in juvenile (6 months) and adolescent monkeys (18months). Interestingly, the magnitude of the recognition memory deficit after neonatal PRH lesions was milder than following adult-onset PRH lesions^103,104^, suggesting functional compensation may have occurred following the early perturbation. It is possible that other brain structures compensate for the loss of PRH-supported familiarity processes in the event of an early PRH malfunction. There is evidence of increased recovery of sensorimotor and visual function following early injury (for review see^111^), but comparably fewer studies in cognitive systems^112–115^.

Taken together, this work has enhanced our understanding of the development of recognition memory and of early brain plasticity.

#### Spatial memory:

**Figure 6:**
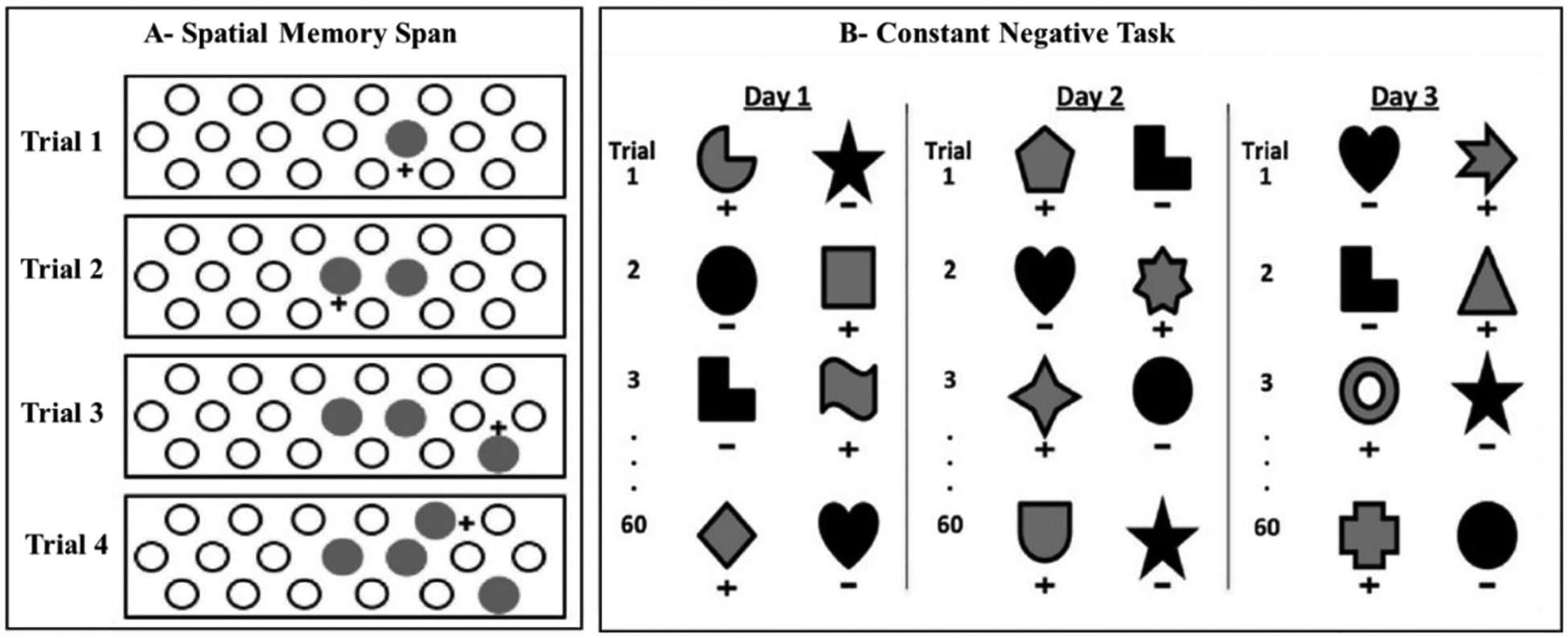
Schematic representation of cognitive tasks. A- Spatial Memory Task (SMS) in which animals must select the newer well covered by a round disk on each trial. ITI intervals lasted 30sec; B- Constant Negative task in which monkeys are given a set of 60 unique discrimination problems on each day and choose between a rewarded novel object (S+ shown in gray) and an unrewarded object that was repeated in each daily session (S- shown in black). A 30-s interval separated each discrimination problem. Adapted from Heuer and Bachevalier (2011); Weiss et al. (2017).

Contrary to its significant contribution to object recognition memory, the PRh plays a limited role in memory for spatial locations^116–122^, especially when delays are kept short^123,124^. Thus, we assessed the effects of neonatal PRH lesions on spatial location memory using the same controls and animals with neonatal PRH lesions as they reached adulthood using a Spatial Memory Span task (SMS, Figure 6A), measuring recognition of spatial locations, and the Delayed Nonmatching-to-Sample (DNMS; Figure 1A), measuring object recognition for comparison. The neonatal PRH lesions had no impact on spatial memory, though they did impair performance on the DNMS task when the delays were extended from 30 s to 600 s^104^. The dissociation between the sparing of spatial memory together with the loss of object recognition memory after neonatal PRH lesions is similar to that described when the PRH damage occurs in adulthood^116,121,125^, suggesting that the PRH is a cortical structure critical for the normal development of processes supporting object memory but not spatial memory.

### Neonatal Perirhinal Lesions and Perceptual Ability versus Familiarity Judgment

Previous studies in adult monkeys with PRH lesions report a mild perceptual impairment when test stimuli were black and white (B&W), or had overlapping/similar features^36–39,126^, suggesting that this cortical area may also contribute to higher-order visual processes. To test the impact of the neonatal-PRH lesions on perceptual abilities, and to confirm a specific impairment in familiarity judgement, we conducted a set of studies in the same monkeys with neonatal PRH lesions discussed above. First, we re-tested the animals on a version of the VPC-object-delays task (See Figure 4) using highly similar black and white (B&W) stimuli. Both Control animals and those with neonatal PRH lesions performed well on the VPC task with similar B&W objects at short (10s) delays, but neonatal PRH animals had significantly lower novelty preferences than controls when the delays were extended beyond 30s^127^. Given the normal levels of novelty preference after the shorter delay, coupled with delay-dependent reduced novelty preference, these data suggest that the neonatal PRH animals had perceptual abilities within the normal range, indicating normal visual perception but displayed recognition memory impairment.

In parallel, the same animals were also tested on a task measuring the progressive accumulation of familiarity signals, the Constant Negative task^128^ (Figure 6B). This experiment was inspired by the observation that, compared with adult-onset PRH lesions, monkeys with neonatal PRH lesions exhibited a partial sparing of recognition memory when measured using VPC, but not when measured using DNMS^103,104^.

A possible explanation for this difference was that the DNMS task used a shorter familiarization time (usually 3–7s) than the VPC task (30s), and therefore that the recognition memory sparing observed with the VPC task could be due to a longer opportunity to become familiar with the sample stimuli. To confirm this, we administered an object discrimination task that measured the rate at which neonatal PRH-operated animals became familiar with objects^128^. The results revealed clear group differences in learning curves, with control animals having significantly steeper slopes than the neonatal PRH-operated animals^127^, suggesting a slower ability to familiarize. Importantly, an alternative interpretation is that, rather than completing the Constant Negative task using novelty-guided strategies, the neonatal PRH-operated monkeys learned to avoid incorrect, unrewarded objects using “habit” based strategies. Habit learning was tested in the same animals using a Concurrent Discrimination task (as in^129^), and comparisons of errors on both tasks indicated that control animals made fewer numbers of errors on the Constant Negative task than the Concurrent Discrimination task, whereas neonatal PRH-operated animals made similar numbers of errors on both. These data suggest that the neonatal PRH-operated animals may have developed a behavioral strategy that relied more heavily on habit systems to compensate for their impaired recognition memory.

## Neonatal Perirhinal Lesions and Working Memory Tasks with High-proactive Interference

**Figure 7:**
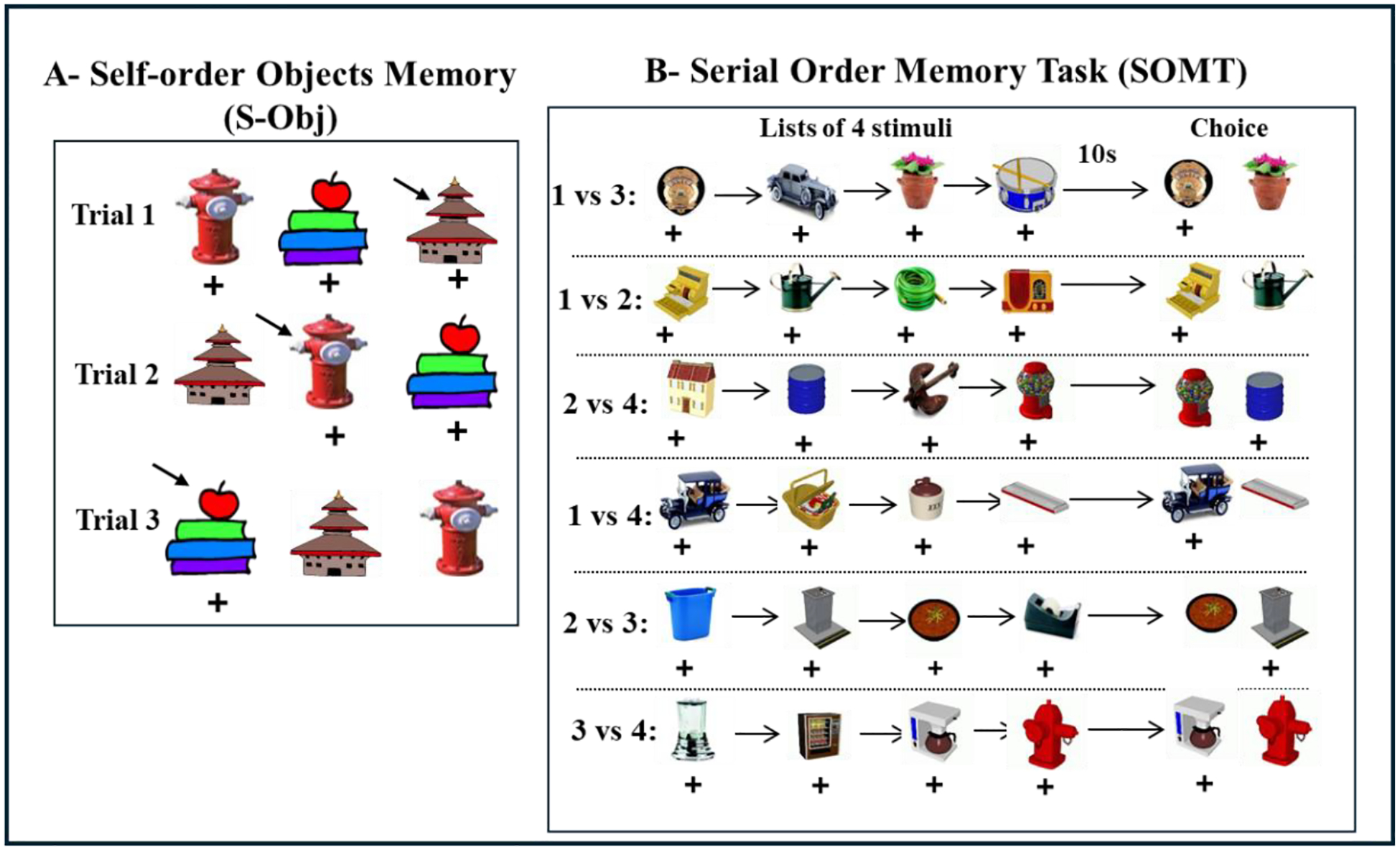
Schematic representation of tasks. A- S-Obj Task measures the animal’s ability to self-monitor its choices. Daily session includes 3 trials. In the first trial, animal can select one of the 3 objects. On trial 2, animal can select one of the two objects not selected in Trial 1 and in trial 3, animals can select the last object not selected in the previous trial. The same 3 objects are used for all trials/sessions. B- SOMT measures animal’s ability to monitor the order of the objects in a list of 4 objects. On each trial, animal is presented with a list of 4 rewarded objects one by one and after a 10 s delay, it is presented with a choice either between objects 1 vs 3, 1 vs 2, 2 vs 4, 1 vs 4, 2 vs 3, or 3 vs 4 and must select the object that appears first in the list. A new list of objects is provided for each trial. Described in detail in Petrides (1995); Weiss et al. (2016), respectively.

In adulthood, the same control animals and those with neonatal PRH lesions were evaluated on three object-based working memory tasks that targeted distinct cognitive demands^130^. One task emphasized simple maintenance (Session-Unique Delayed Nonmatching-to-Sample; SU-DNMS), which is like the DNMS shown in Figure 1A, but only two stimuli are used on each trial and serve either as the familiar or the novel stimulus. The other two WM tasks required maintenance plus monitoring of temporal item order: the Object Self-Order (S-OBJ, Figure 7A) and the Serial Order Memory Task (SOMT. Figure 7B).

Monkeys with neonatal PRH damage showed a deficit on SU-DNMS when they were initially tested with a short delay of 5 seconds, but they performed normally when re-tested and the delay extended to 30 seconds, indicating that this impairment was transient and that they were able to perform this task at a level comparable to controls with extended opportunities for practice. Similarly, performance on the SOMT was unaffected, indicating preserved ability to track the temporal sequence of items in working memory following these early lesions. In contrast, the neonatal PRH-operated animals made significantly more errors on the S-OBJ task, most notably reflected in a marked rise in perseverative responding. Together, the evidence from the SU-DNMS, S-OBJ, and SOMT tasks indicated that early damage to the PRH impacted the development of mechanisms that help resolve proactive interference and/or control perseverative responding, but spared mechanisms supporting object representations in working memory. Due to increased brain plasticity during development, it is possible that other brain areas, such as the hippocampus or temporal visual cortical areas could be recruited to support object representations in working memory in the absence of a functional PRH. Thus, although neuroimaging and electrophysiological data clearly indicate that in adult subjects the PRH is involved in working memory processes, in the absence of a functional PRH during infancy, the data suggest that these cognitive processes may develop differently and rely on other brain areas.

### Neonatal Perirhinal Lesions and Cognitive Flexibility

**Figure 8:**
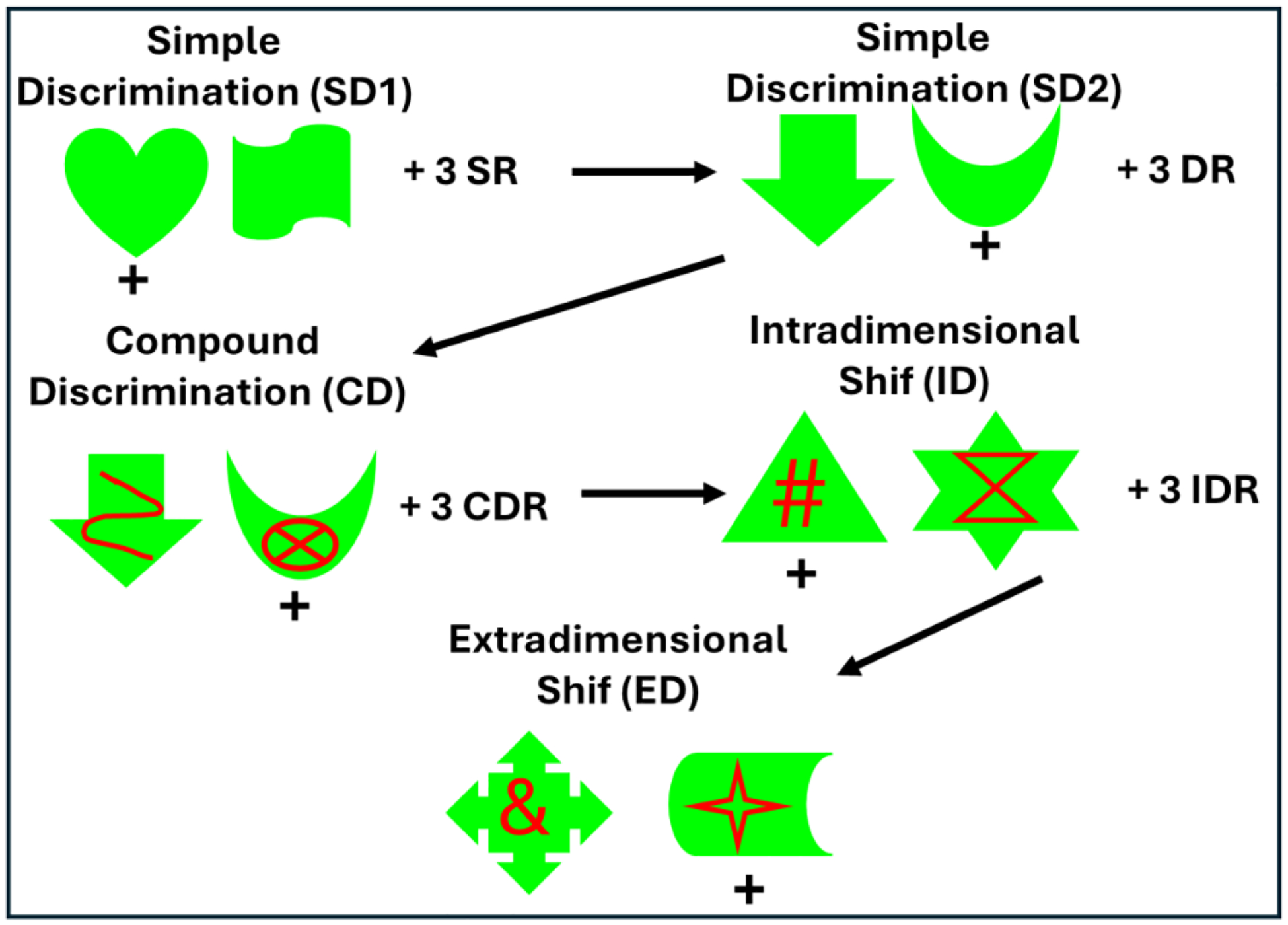
IntraDimensional/ExtraDimensional (ID/ED) task measures behavioral inhibition and cognitive flexibility. The animal first learns two simple discrimination problems (SD1, SD2) followed by 3 reversals of the second problem (SR). The animal learns a compound discrimination problem (SC) with red lines occurring on top of the green shapes of the last discrimination but continues to respond to the shape, followed by 3 reversals (CDR). In the intradimensional shift (ID), new shapes and lines form a new discrimination problem during which the animal continues responding to the shape, followed again by 3 reversals (IDR). Finally, in the extradimensional shift (ED), a new discrimination problem is presented with new shapes and lines, but now the animal needs to respond to the lines. Described un details in Weiss et al. (2019).

As described above, the performance patterns of neonatal PRH-operated animals on object-based working memory tasks suggested that the early PRH damage disrupted the development of mechanisms that resolve proactive interference. To further test whether this vulnerability reflected broader impairments in inhibitory control or difficulty shifting behavioral strategies, we evaluated the same neonatal PRH-operated monkeys and Control animals on an Intradimensional–Extradimensional (ID–ED) set-shifting task in adulthood^131^. This paradigm has proved valuable for distinguishing between two components of executive function: (1) *reversal learning*, which depends heavily on inhibiting previously reinforced responses, and (2) *cognitive flexibility*, which requires shifting attention away from a previously relevant stimulus dimension toward a newly relevant one. In the early phases of the task (simple discriminations, compound discriminations, and reversals; Figure 8) the neonatal PRH-operated monkeys performed comparably to controls, indicating intact basic stimulus–reward learning and relatively preserved behavioral inhibitory control. However, when required to shift attention across stimulus dimensions, their performance declined sharply. Unlike control animals that rapidly adapted to the new rule, neonatal PRH-operated monkeys showed persistent perseverative responding, continuing to rely on the previously learned dimension despite repeated negative feedback. This selective impairment at the extradimensional shift stage pointed to a deficit in cognitive flexibility rather than a generalized learning or memory problem^131^.

Together with the working memory findings^130^, these results indicate that early PRH removal alters the maturation of neural systems that support flexible, interference-resistant behavior. The sparing of reversal learning suggests that the basic ability to suppress previously rewarded responses remains largely intact, whereas the capacity to reconfigure behavior in response to changing task demands is compromised. Importantly, the deficit in cognitive flexibility following neonatal PRH damage contrasts with the minimal cognitive flexibility effects reported after broad medial temporal lobe lesions in adulthood, including damage to the PRH^91^.

Given the dense reciprocal connections between the PRH and ventrolateral prefrontal cortex^19,22,24,132,133^, an area known to undergo protracted postnatal maturation, one interpretation of these results is that the neonatal PRH lesions disrupted the developmental scaffolding that normally supports the emergence of flexible executive control. These observations are consistent with the broader pattern in this cohort, in which recognition and working memory show partial functional compensation, but higher-order control processes remain particularly vulnerable to early PRH dysfunction. Taken together, these findings indicate that the absence of normal PRH projections to ventrolateral and medial PFC during early development may interfere with the establishment of prefrontal networks necessary for adaptive behavioral control, leading to long-lasting impairments in cognitive flexibility even when working processes remain relatively preserved.

## Perirhinal Cortex and Human Clinical Disorders

Finally, the PRH has now been shown to be a prominent neural marker of the prodromal phase in at least three human clinical neurodevelopmental disorders, epilepsy, Alzheimer’s disease, and schizophrenia, briefly reviewed below.

### Temporal Lobe Epilepsy

Recurrent unprovoked seizures are the hallmark of epilepsy, and medial temporal lobe epilepsy (TLE) is the most common type of medically intractable focal epilepsy in adolescents and adults that necessitates surgical evaluation. Ninety years of studies on TLE patients with either medial temporal lobe lesions or electrical stimulations have strengthened the theoretical view of the respective role of PRH, ERH and hippocampus in memory processes reviewed above (see for review^134,135^).

Neuroimaging studies have documented volume loss of the anterior temporal lobe, temporal neocortical gray and white matter, and more refined analyses have also revealed volumetric changes of the hippocampus, PRH, and ERH^136^. Interestingly, a small subset of individuals with TLE consistently experience “déjà-vu” phenomenon during the aura of their seizures (see for review^137,138^). This experiential phenomenon was recognized very early by the pioneering work of Jackson^139,140^ and subsequently followed by Penfield^141^ and Gloor^137^. Déjà-vu phenomenon is defined as an alteration of consciousness characterized by memory-like hallucinations, and/or a feeling of familiarity, with an epileptiform activity localized to the anterior parahippocampal region^142–146^. The link between déjà-vu and medial temporal region in TLE is of particular interest in the context of the role of that brain region in recognition memory.

As reviewed above, the flow of information about items from PRH to ERH, through the hippocampal fields, and back to ERH and PRH provides a reverberatory circuit that, in normal conditions, plays a crucial role in the processing of declarative memory. Under pathological conditions, however, this neural loop tends toward excessive propagation and loop-gain amplification, which is a hallmark of TLE^147^. Concurrently, many current models of recognition memory offer a critical distinction between familiarity assessment and recollection (see for review^54^). Familiarity assessment involves evaluating memory strength of an item independent from recovery of contextual detail about a specific past encounter and has been proposed to depend specifically on computations performed in PRH, and not in the hippocampus^148–150^. Recollection, by contrast, involves direct recovery of contextual details about a specific prior encounter with an item and has been linked specifically to hippocampal functioning^54,151^. When considered within this model, part of the typical déjà-vu experience in the aura of TLE has been characterized as a static sense of familiarity (e.g., Gloor^137^). Accordingly, the phenomenon may reflect, at its core, an erroneous familiarity signal that is generated by abnormal activity in PRH, or perhaps combined PRH-ERH (see also^152^). Preserved recollection and metacognitive inferences may produce the subjective sense of inappropriateness for this familiarity signal. Considered together, the evidence reviewed hints that TLE patients who experience déjà-vu in the aura of their seizures may present with lasting interictal behavioral impairments that are specific to familiarity assessment.

Yet, there is still very little direct evidence for this proposal, but provocative results exist. Bowles and colleagues^45^ reported a single TLE case study presenting with a surgical removal of the MTL including the PRH but sparing the hippocampus showing impaired familiarity with preserved recollection. Furthermore, Martin and colleagues^153^ demonstrated selective familiarity deficits sparing recollection in patients with unilateral TLE experiencing déjà vu that contrasted with the broader pattern of recognition-memory impairments present in a control group of unilateral TLE patients without déjà-vu. Interestingly, medial temporal lobe structures were less broadly affected in TLE patients with déjà-vu than in TLE patients without déjà-vu, with a trend for more focal volume reductions in the PRH+ERH cortices of those patients with déjà-vu. Additional evidence for the distinctive contribution of the PRH vs hippocampus in the familiarity feeling experienced during déjà-vu episodes was provided by numerous brain stimulation studies of TLE patients^135,143^. The results indicate that electrical stimulation of the amygdala and hippocampus induced reminiscences of episodic memories, whereas stimulations of the PRH-ERH lead mostly to familiarity and semantic memories, in other words to memories devoid of contextual information. Fernández and Tendolkar^154^ hypothesized that the PRH-ERH could be a gatekeeper to the declarative memory system and acts as an adaptable interface between the neocortex and the hippocampus. For example, an early PRH familiarity signal could trigger a source retrieval process for recollection in the hippocampus, which could in turn recruit PRH representations during episodic memory processes^50,51^. Furthermore, the PRH has prominent and convergent projections from sensory polymodal cortical areas and prominent interconnections with other medial temporal lobe structures and projects back to a wider extent of the cortex than it receives input from, including some area that do not project to it at all^19,155^. To sum, in addition to providing critical neuroanatomical information for the treatment of intractable TLE, the studies also prompt a theoretical interest for the scientific literature at large regarding the neural substrate of memory processes.

### Alzheimer’s Disease

Alzheimer’s disease (AD) is one of the most prominent degenerative diseases and the major cause of elderly disability. It typically progresses from a long preclinical asymptomatic phase to mild cognitive impairment (MCI) that becomes incurable cognitive impairment in the transition to AD stage^156^. Thus, identifying the earliest signs of AD has sparked a critical interest in the field as this will allow the initiation of treatments as early in the disease progression as possible^157,158^. Neuropathological brain changes associated with AD, namely β-amyloid (Aβ) plaques and neurofibrillary tangles (NFT), are thought to begin years before clinical symptoms become evident. In contrast to β-amyloid plaques, NFT are more strongly correlated with cognitive deficits^159^ and progress in a hierarchical manner throughout the brain in typical AD^160^. This continuous accumulation of NFT is strongly related to loss of neurons^159,161^ and was shown to be causally associated to cerebral atrophy in affected regions^162^.

The most obvious clinical symptom of AD is the progressive loss of declarative memory. Therefore, a large emphasis has been placed to identify the integrity of hippocampus and related medial temporal cortical areas in AD, due to their critical role in declarative memory^3,108,149,163^. With the advent of MRI tools and the growing use of imaging in clinical settings, the earliest AD-related changes on structural MRI are usually seen in the medial temporal lobe structures and notably the hippocampus (see for a detailed review^164^). Hence, the hippocampal volume is reduced by 10–15% in MCI patients and by 40% in clinical AD patients compared to elderly controls (for review see^165–168^). Rate of hippocampal atrophy has been shown not only via automated and manual segmentation on structural MRI, but with additional neuroimaging tools, such as 3-D dimensional surface shape measures and metabolic neuroimaging, and is associated with changes in cognitive performance^169–171^. In both, MCI and AD, surface inward-deformation of the hippocampus is more prominent in its anterior portion as well as its lateral border (likely associated with CA1 atrophy)^172^.

Thus, hippocampal alteration was viewed as major topographical marker linking molecular pathology of AD to the clinical and cognitive decline. Nevertheless, in addition to hippocampal atrophy, Pennanen and colleagues^173^ showed that ERH volume loss was the dominant structural feature at the stage of MCI, followed by profound macroscopic hippocampal atrophy in patient with clinical AD. Positron Emission Tomography (PET) studies have also revealed that hypometabolism in the posteromedial association cortex is among the earliest brain areas containing Aβ plaques^174–176^ and may reflect a disconnection of the medial temporal cortical areas from the hippocampus. Thus, the ERH and PRH are some of the earliest cortical changes, prior to the hippocampal atrophy and preceding the cognitive impairment^173,177,178^. Utilizing the U-Net framework, Henzen and colleagues^179^ recently confirmed that the medial PRH is one of the earliest regions affected by neurofibrillary tau pathology, which approximately corresponds to Brodmann area 35 (see Figure 3A). This neurofibrillary tau pathology spreads to the medially located ERH and eventually to the hippocampus, and throughout the brain. Both automated MRI segmentation, optical imaging techniques to illuminate large-scale structures in postmortem tissue at a micron-scale resolution, as well as transcriptional regulation of the gene for synaptic protein, are becoming promising tools to establish early markers of PRH pathology. The importance of future longitudinal studies using these methodologies together with neuropsychological testing using tasks specifically assessing recognition memory processes (familiarity versus recollection), as well as working memory, and cognitive flexibility will enhance diagnostic precision and pave the way for early, targeted intervention strategies. Furthermore, as for epilepsy reviewed above, the clinical data may strengthen the proposal for the distinctive role of PRH and hippocampus in recognition memory processes.

### Schizophrenia

Schizophrenia is a chronic neurodegenerative psychiatric disorder with an early onset in young adulthood reflecting the interplay of genetics and the environment^180–182^. The disease characteristically evolves from a premorbid phase in which the clinical phenotype is not, or only partially, expressed through a series of stages culminating in the syndromal manifestation (hallucination, delusion, speech and behavior disorder, apathy, social withdrawal, and cognitive impairment) meeting diagnostic criteria and constituting a first episode of a psychotic disorder^183^. The subsequent course varies markedly based on illness severity, adequacy of treatment and environmental, including social, factors. As with other progressive disorders of the brain, such as Alzheimer’s disease (see above), early detection and treatment during prodromal stages, when the disease is restricted to relatively confined areas of the brain, has emerged as an important therapeutic strategy in alleviating symptoms and disease modification. Previous research indicates that people with schizophrenia exhibit impairments in declarative memory and their severity correlates with the magnitude of structural abnormalities within the medial temporal lobe structures^184–186^, including the hippocampus together with its neighboring cortical areas, ERH and PRH cortex. Consistent with this idea, structural changes in the hippocampus (reduced volume, presence of cellular and molecular abnormalities, and abnormal activity; see^187^ for a review) and abnormal volumes of the parahippocampal gyrus (ERH and PRH)^188–190^ have been found in schizophrenia patients. Furthermore, a resting state functional connectivity study showed alterations characterized by increases and decreases in the strength of the positive connectivity between the PRH and ERH and the hippocampal subregions when comparing patients with schizophrenia with healthy subjects^191^. Concurrently, schizophrenia patients experience difficulties in recognition tasks with both familiarity and recollection deficits reported in some studies, but only recollection deficits in others, suggesting multi-focal medial temporal lobe dysfunction^192^. Thus, both the medial temporal lobe neuropathological findings as well as the memory deficits in schizophrenia offer significant evidence to implicate a role of the hippocampus and adjacent cortical areas in schizophrenia. However, additional research is necessary. The integration of anatomical and functional neuroimaging tools coupled with direct assessment of recognition memory processes to further characterize specific recollection versus familiarity deficits in the schizophrenia prodrome phase will likely provide critical neuroanatomical markers for better targeted treatment of memory impairments.

## Conclusions

Significant advances have been made in the last decade on the specific role played by the medial temporal lobe structures in memory processes. Although extensive literature exists on the prominent function of the hippocampus in recollection, more recent emphasis was placed onto one of the medial temporal cortical areas surrounding the hippocampus, namely the PRH. In this review we showed that the PRH plays a critical role in recognition memory, particularly familiarity judgements rather than recollection, and in object memory rather than spatial memory. In addition, the PRH modulates anxiety as well as impulse control and behavioral flexibility. A series of developmental studies in nonhuman primates also indicated that anatomically and functionally the perirhinal cortex matures early in infancy, unlike the more protracted maturation of the hippocampus in adolescence, and PRH damage in infancy impacts object recognition memory at an early age. Further findings indicated that as animals with neonatal perirhinal damage reached adulthood, they displayed some functional compensation of object recognition and working memory but were severely impaired in higher-order executive processes such as cognitive control and flexibility. Interestingly, the discovery of the role of PRH in familiarity judgements instead of recollection is now well documented in several clinical neurodevelopmental disorders associated with memory disorders and cognitive impairments and is providing critical markers for early diagnoses and treatment.
